# Aberrant Expression of ADARB1 Facilitates Temozolomide Chemoresistance and Immune Infiltration in Glioblastoma

**DOI:** 10.3389/fphar.2022.768743

**Published:** 2022-02-01

**Authors:** Can Lu, Xi Chen, Yuanliang Yan, Xinxin Ren, Xiang Wang, Bi Peng, Yuan Cai, Qiuju Liang, Zhijie Xu, Jinwu Peng

**Affiliations:** ^1^ Department of Pathology, Xiangya Hospital, Central South University, Changsha, China; ^2^ Department of Pharmacy, Xiangya Hospital, Central South University, Changsha, China; ^3^ Key Laboratory of Molecular Radiation Oncology of Hunan Province, Xiangya Hospital, Central South University, Changsha, China; ^4^ Department of Pathology, Xiangya Changde Hospital, Changde, China; ^5^ National Clinical Research Center for Geriatric Disorders, Xiangya Hospital, Central South University, Changsha, China

**Keywords:** glioblastoma, temozolomide, ADARB1, akt, immune

## Abstract

Chemoresistance, especially temozolomide (TMZ) resistance, is a major clinical challenge in the treatment of glioblastoma (GBM). Exploring the mechanisms of TMZ resistance could help us identify effective therapies. Adenosine deaminases acting on RNA (ADARs) are very important in RNA modification through regulating the A-to-I RNA editing. Recent studies have shown that ADARs regulate multiple neurotransmitter receptors, which have been linked with the occurrence and progress of GBM. Here, data from several bioinformatics databases demonstrated that adenosine deaminase RNA specific B1 (ADARB1), also named ADAR2, was upregulated in both GBM tissues and cells, and had the prognostic value in GBM patients. Moreover, ADARB1 was found to be involved in AKT-mediated TMZ resistance in GBM cells. The KEGG analysis of ADARB1-associated co-expressed genes showed that ADARB1 was potentially involved in the mitochondrial respiratory chain complex. TISIDB and GEPIA databases were further used to analyze the role of ADARB1 in tumor-immune system interactions in GBM. These findings deepened our understanding of the function of ADARB1 in tumorigenesis and therapeutic response in GBM.

## Introduction

Glioblastoma (GBM) is the most malignant and aggressive brain tumor in adults, with a median survival of 12–15 months (Chen et al., 2019; Chen et al., 2020). The tumor cells of GBM show an infiltrative growth pattern into surrounding normal tissues, which is hard to be eliminated by surgical resection, resulting in a high recurrence rate (Azzarelli, 2020). Nowadays, temozolomide (TMZ) is the standard chemotherapy drug for GBM treatment, but the median survival time has not been notably prolonged. Meanwhile, most GBM patients could gradually develop resistance to TMZ treatment (Yan et al., 2019). Therefore, improving the TMZ chemosensitivity is a very urgent issue in the clinical treatment of GBM.

Adenosine deaminases acting on RNA (ADARs) are important in post-transcriptional RNA modification, especially A-to-I RNA editing (Li and Church, 2013). A-to-I RNA editing regulates the key functions and characteristics of multiple neurotransmitter receptors, which are known to play a role in the occurrence and progress of glioma (Seeburg, 2002). The abnormalities of ADARs are also closely related to multiple diseases, such as viral infections, metabolic diseases, and tumors (Macbeth et al., 2005; Paz et al., 2007). Previous studies have reported that A-to-I RNA editing mediated by adenosine deaminase RNA specific B1 (ADARB1) is significantly impaired in GBM tissues and cells (Tomaselli et al., 2015). Similarly, Tomaselli et al. found a significant loss of ADARB1-editing activity in GBM tissues compared with normal brain (Tomaselli et al., 2013). Activation of ADARB1-editing activity suppresses cell growth through increasing CDC14B signaling (Galeano et al., 2013). However, the molecular mechanism underlying the biological roles of aberrant ADARB1 in GBM chemotherapy and its biological significance has not been fully validated and elucidated.

Protein kinase B (AKT) plays an important role in tumorigenesis. A high level of phosphorylated AKT (p-AKT) has been reported to be associated with poor prognosis in GBM patients (Xue et al., 2015). Inhibitors targeting the AKT/GSK3β pathway seem to have strong therapeutic potential (Liu et al., 2020). Nevertheless, the modest therapeutic effect exerted by these compounds in clinical trials suggests that they might be useful in combination therapy rather than in single-agent therapy. Clinical trials of AKT/GSK3β pathway inhibitors combined with TMZ, radiotherapy, and bevacizumab are in progress (Odia et al., 2016).

GBM is also a profoundly heterogeneous tumor that facilitates immune evasion. Immunotherapeutic strategies for GBM have a long history, including immune stimulation, antibody-mediated immunotherapies, adoptive cellular immunotherapies, and vaccines (Tamura et al., 2020; Yan et al., 2020). Previous studies have reported that several factors associated with the immune response have changed significantly in GBM, resulting in tumor immune evasion (Crane et al., 2014). In addition to the standard-of-care treatments commonly used for GBM patients (e.g., surgery, chemotherapy, and radiotherapy), immunotherapy is increasingly recognized as a particular form of tumor treatment that stimulates the immune system and activates specific immune cells to attack tumor cells. However, the immune microenvironment of GBM is rarely discussed in comprehensive studies, which needs to be further explored.

Our study aims to investigate the molecular mechanism underlying the aberrant expression of ADARB1 in GBM biology. In our study, ADARB1, also named ADAR2, was found to influence the TMZ chemotherapy response. In addition, higher expression of ADARB1 was identified in GBM tissues and cell lines, which was associated with poor prognosis.

## Materials and Methods

### Data Acquisition and Reanalysis Using Different Bioinformatics Tools

The effects of ADARB1-related chemoresistance of GBM were investigated using multiple bioinformatics databases. The databases are listed in Supplementary Table S1. Several GBM datasets were downloaded from the GEO database (https://www.ncbi.nlm.nih.gov/gds) (Table 1). Oncomine is a web-based data-mining platform aimed at facilitating the expression analyses of selected genes across multiple datasets (Rhodes et al., 2004). Thus, from the Oncomine database, we used two GBM transcriptome microarray datasets, GSE4536 (Lee et al., 2006) and GSE4209 (Ward et al., 2006), to evaluate the expression of ADARB1 in GBM patients. Another TMZ therapeutic transcriptome microarray dataset is GSE80729 (Wu et al., 2018). Chemotherapy-related datasets analyzed by using DRUGSURV and PrognoScan databases, including GSE13041 (Lee et al., 2008), GSE4412 (Freije et al., 2004), and GSE4271 (Norton et al., 2008), were used to analyze the impacts of ADARB1 expression on the chemotherapy response of GBM.

**TABLE 1:** The primary characteristics of 6 GEO datasets on gene expression profiling *via* microarray.

GEO^a^ datasets	Platform	Tissues		Cells	References
		Cancer	Normal		
GSE4536	GPL570	98 (Glioblastoma)	30 (Neural stem cell)	—	(18)
GSE4290	GPL570	157 (Glioblastoma)	23 (Brain)	—	(19)
GSE80729	GPL10558	—	—	U87 siCtrl *vs* TMZ	(21)
GSE13041	—	267 patients	—	—	(22)
GSE4412	GPL96/97	74 patients	—	—	(23)
GSE4271	GPL96/97	77 patients	—		(24)

a GEO, Gene Expression Omnibus datasets.

Next, 19,915 ADARB1-associated co-expressed genes in GBM pathology were downloaded from the cBioportal database (Gao et al., 2013). We then constructed a protein-protein interaction (PPI) for these co-expressed genes from the STRING database, which integrates known and predicted PPI networks from many organisms (Szklarczyk et al., 2017; Wu et al., 2020a). Afterward, we applied the Cytoscape software (Wu et al., 2019) to visualize the PPI network of these co-expressed genes. Additionally, we used WebGestalt to analyze the GO and KEGG pathways of co-expressed genes with ADARB1 in GBM (Huang et al., 2009).

The distributions of immune cells related to ADARB1 expression are shown in the bubble diagram by ssGSEA analysis of Xiantao Tool (https://www.xiantao.love/products). The TISIDB database is an integrated repository portal for tumor-immune system interactions (Ru et al., 2019). We also used the TISIDB database to analyze the association between ADARB1 expression, tumor-infiltrating lymphocytes (TILs), immunomodulators, and other immune molecules in GBM patients.

The GEPIA database is a newly developed interactive web server. Through a standard pipeline, analysis of RNA sequencing expression data of 9,736 tumors and 8,587 normal samples from the TCGA and the GTEx projects can be achieved (Li et al., 2021). In this database, Li and his colleague downloaded the TCGA tumor/TCGA normal/GTEx datasets. Then, the log-normalization was performed to analysis the differential expression level of candidate genes. Thus, we used the GEPIA database to analyze the expression association between ADARB1 and immune checkpoints in GBM patients.

### Cells and Reagents

T98G-R and U118-R TMZ-resistant glioma cell lines and their parental cell lines T98G and U118 were established and cultured as previously described (Dai et al., 2017; Yan et al., 2019). The AKT inhibitor MK2206 was purchased from Selleck Chemicals and dissolved in dimethylsulfoxide (DMSO) (Sigma). The exposing concentrations of TMZ, MK2206 were 200 and 5 mM, respectively.

### Transient Transfection

Small interfering RNA (siRNA) targeting ADARB1 (si-ADARB1,50-CAGGCACAGAUGUUAAAGATT-30) was purchased from Genepharma (Suzhou, China), and a scrambled siRNA (si-Ctrl,50-UUCUCCGAACGUGUCACG UTT-30) was used as the negative control. Transfection was performed using Lipofectamine 3,000 reagent (Invitrogen, Carlsbad, CA, United States) according to the manufacturer’s protocol. After the indicated incubation times, the cells were harvested and subjected to protein extraction or other cellular experiments.

### RNA Extraction and Quantitative PCR

Altogether 10 pairs of formalin-fixed, paraffin-embedded (FFPE) specimens of primary and recurrent GBM tumor tissues were collected from Department of Pathology, Xiangya Hospital (Changsha, China). The ethics of our study has been approved by the Ethical Committee of Xiangya Hospital of Central South University. The ethical approval number is 202,110,206. Total RNA was extracted from FFPE tissue specimens using PureLink FFPE RNA Isolation Kit (K156002; Invitrogen, United States) according to the manufacturer’s protocol, followed by cDNA synthesis using a PrimeScript RT reagent kit (Takara, China). The qPCR was conducted with iTaq Universal SYBR green Supermix (Bio-Rad, United States). The forward and reverse primer sequences are as follows: CXCL12: 5′- ATT​CTC​AAC​ACT​CCA​AAC​TGT​GC-3′ and 5′-ACT​TTA​GCT​TCG​GGT​CAA​TGC-3’; B2M: 5′- GAG​GCT​ATC​CAG​CGT​ACT​CCA-3′ and 5′-CGG​CAG​GCA​TAC​TCA​TCT​TTT-3’; TABPB: 5′- TGG​ACC​GGA​AAT​GGG​ACC​T-3′ and 5′-CCC​CAG​AAG​GGT​AGA​AGT​GG-3’.

Total RNA extracted from glioma cell lines was using TRIzol reagent (Invitrogen, Carlsbad, CA, United States) according to the manufacturer’s protocol. Afterward, total RNA was reverse transcribed to cDNA using the PrimeScriptTM RT reagent kit (Takara, Dalian, China). The qPCR assay was performed using iTaq Universal SYBR Green Supermix (Bio-Rad, United States), with b-actin as the internal control. The forward and reverse primer sequences are as follows: β-actin: 5′-CAT​GTA​CGT​TGC​TAT​CCA​GGC-3′ and 5′-CTC​CTT​AAT​GTC​ACG​CAC​GAT-3’; ADARB1: 5′-GTG​AAG​GAA​AAC​CGC​AAT​CTG​G-3′ and 5′-CAG​GAG​TGT​GTA​CTG​CAA​ACC-3’. Relative expression levels were calculated by the 2^-△△CT^ method. All reactions were run at least three times.

### Western Blot

Protein extraction was performed as previously described (Wu et al., 2020a). Afterward, protein samples were resolved by SDS-PAGE, transferred to polyvinylidene difluoride membrane, and hybridized with antibodies specific to ADARB1 (22248-1-AP, Proteintech), AKT (9272s, Cell Signaling Technology), p-AKT (4,060, Cell Signaling Technology), GAPDH (60004-1-Ig, Proteintech). Protein bands were visualized in the ChemiDoc XRS system (Bio-Rad, Berkeley) using the HRP substrate chemiluminescence reagent (Millipore, United States).

### MTS Assay

Cell viability and cell survival were analyzed by MTS assay. After 24 h of transfection, cells were re-seeded in 96-well plates at a density of 3 × 10^3^/well. The value of cell survival was detected at 490 nm using a microplate reader (BioTek, Winooski) after a 1-h incubation with MTS solution (Dojindo, Kumamato). The experiments were repeated at least three times, and each experiment was performed at least twice.

### Statistical Analysis

Statistical analysis was performed with SPSS 12.0 software (IBM Analytics). All experiments were performed at least three times, and the mean ± SD was subjected to Student’s t-test. Kaplan-Meier analysis was performed to analyze survival rates for GBM. The differential mRNA expression between cancer and non-cancer tissues or between the control group and treatment group were analyzed using Student’s t-test. The associations between ADARB1 expression and clinicopathologic characteristics in GBM patients were assessed using the Kruskal-Wallis rank test or the Mann–Whitney U test. Correlations between genes were analyzed using Spearman’s correlation coefficient. ∗*p* < 0.05, and ∗∗*p* < 0.01 were defined as statistically significant.

## Results

### ADARB1 is Upregulated in GBM and impacts the TMZ Treatment Outcomes

**FIGURE 1:**
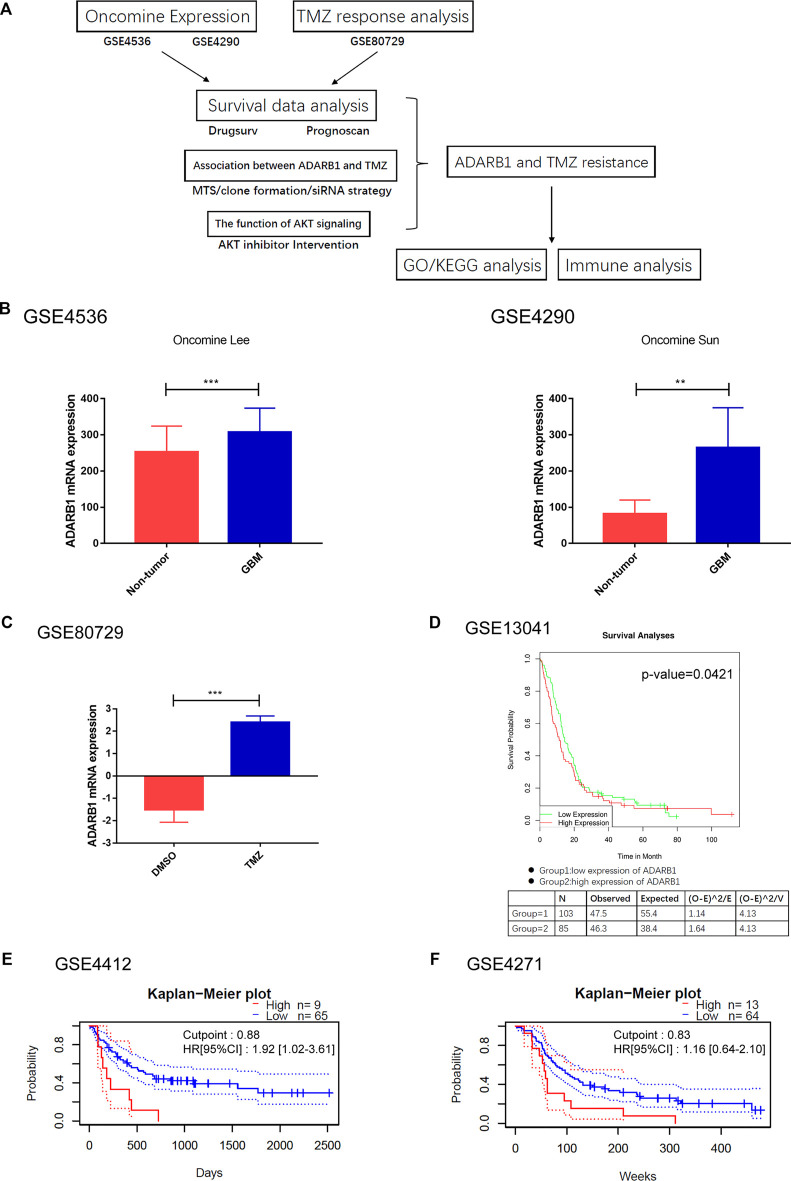
ADARB1 upregulated in GBM. **(A)** The flowchart of the whole analysis process. **(B)** The mRNA expression of ADARB1 in GBM and noncancerous tissues were analyzed using the Oncomine database. Data performed with mean ± SD were subjected to Student’s t-test. **(C)** The mRNA expression of ADARB1 in GBM cells treated with DMSO or TMZ. **(D–F)** The relationship between ADARB1 expression and OS, analyzed from GSE13041, GSE4412 and GSE4271 databases. In GSE13041, the red line refers to the high expression level of ADARB1 and the green line refers to the low expression level of ADARB1. In GSE4412 and GSE4271, the red line refers to the high expression level of ADARB1 and the blue line refers to the low expression level of ADARB1. The Kaplan-Meier curves were extrapolated from GBM patients underwent chemotherapy.

The flowchart of the whole analysis process was shown in Figure 1A. Firstly, the expression of ADARB1 was analyzed using two independent datasets from Oncomine. The result showed that the mRNA expression of ADARB1 was higher in GBM tissues than that in noncancerous tissues (Figure 1B). In addition, the analysis of GSE80729 revealed that treatment with TMZ significantly upregulated ADARB1 expression, indicating that ADARB1 may influence the treatment outcomes of GBM patients (Figure 1C). Then, the correlation between the ADARB1 expression and patients’ prognosis was analyzed in several GBM datasets using the Kaplan-Meier plotter of Drugsurv (Amelio et al., 2014) and PrognoScan (Mizuno et al., 2009). As shown in Figures 1D–F, high expression of ADARB1 was negatively associated with the prognosis of GBM patients. In addition, given that ADAR1 functions overlap with ADARB1, we wanted to explore the files of ADAR1 in GBM. Unexpectedly, there was no significant change in expression of ADAR1 between GBM tissues and noncancerous tissues (Supplementary Figures S1A,B). And abnormal expression levels of ADAR1 did not affect the prognosis (Supplementary Figure S1C). Collectively, the above results suggested that ADARB1 expression levels were upregulated in both GBM tissues and cell lines, and may influence the tumorigenesis and therapeutic responses in GBM patients.

### ADARB1 is involved in TMZ Resistance of GBM Cells

**FIGURE 2:**
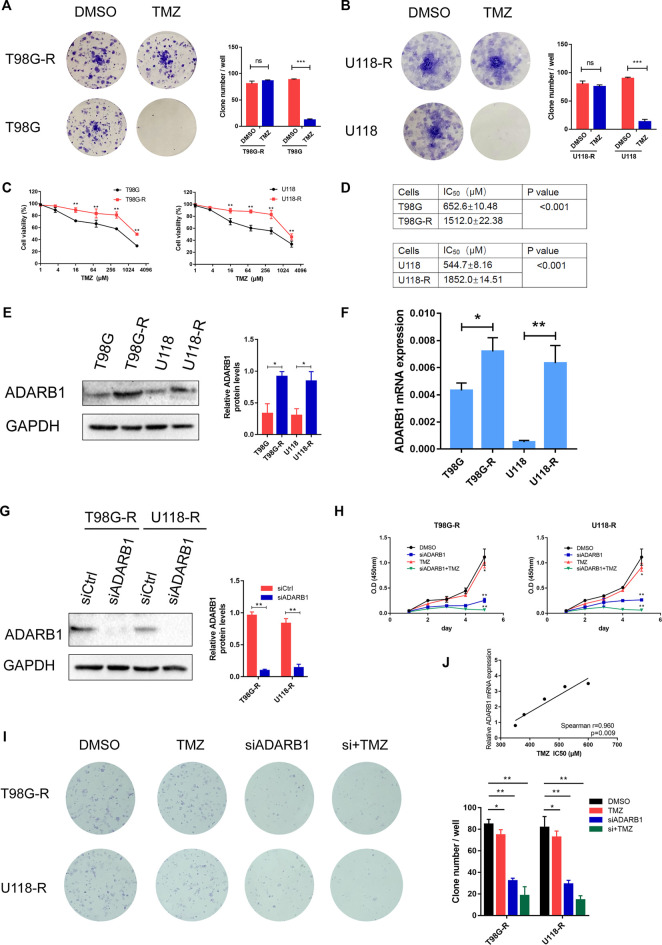
ADARB1 involved in TMZ resistance in glioma cells. **(A,B)** Establishment of TMZ-resistant glioma cells in T98G and U118 cells. Compared with T98G and U118 cells, T98G-R and U118-R cells were remarkably more resistant to the treatment of TMZ in colony formation assay. **(C,D)** Cell viability analysis was performed to evaluate cytotoxicity of TMZ to T98G, T98G-R, U118, and U118-R cells under treatment with the indicated concentrations of TMZ for 72 h **(E,F)** The protein and mRNA expression levels of ADARB1 were significantly higher in T98G-R and U118-R cells, respectively. The results were presented as means ± SD (*n* = 3 for each panel). Statistical significance was concluded at *p < 0.05. **(G)** Western blot for ADARB1 expression in T98G-R and U118-R cells treated with siCtrl or siADARB1. **(H,I)** T98G-R and U118-R cells transfected with siADARB1 or siRNA combined with TMZ treatment were subjected to MTS assays and colony formation assays. **(J)** The correlation between ADARB1 mRNA expression and TMZ IC50 values in five glioma cells was quantified by Spearman’s rank correlation.

To verify the effect of ADARB1 on TMZ chemoresistance, we detected the ADARB1 expression in two TMZ-resistant GBM cell lines (T98G-R and U118-R), which were previously constructed in our lab. The colony formation assay results showed that T98G-R and U118-R cells were more resistant to TMZ than their parental cell lines T98G and U118 cells, respectively (Figures 2A,B). The IC50 values of TMZ-resistant GBM cells (T98G-R: 1512.0 ± 22.38 μM, U118-R: 1852.0 ± 14.51 μM) increased nearly three-fold compared to those of parental GBM cells (T98G: 652.6 ± 10.48 μM, U118: 544.7 ± 8.16 μM) (Figures 2C,D). Meanwhile, ADARB1 expression was significantly higher in T98G-R and U118-R cells than in their parental cell lines at mRNA and protein levels (Figures 2E,F). To further investigate the impacts of ADARB1 on TMZ-resistant behaviors, we used a siRNA-mediated knockdown strategy to inhibit the expression of ADARB1 in TMZ-resistant cells T98G-R and U118-R (Figure 2G). After inhibition of ADARB1 expression, T98G-R and U118-R cells showed decreased cell proliferation rates and fewer colony formations. In addition, when treated with the combination of siRNA and TMZ, the T98G-R and U118-R cells showed a more significant decrease in cell proliferation rate and clone number than when treated with siRNA alone (Figures 2H,I). Meanwhile, we also investigated the effects of ADARB1 knockdown on the parental cell lines T98G and U118, and found an increase in cell proliferation rate and clone number to some extent in the parental cells (Supplementary Figures S2A,B), indicating the opposite effects of ADARB1 in TMZ sensitivity and resistant cells. These conflicting data might be due to the chemotherapy-induced alterations in the gene expression (Arend et al., 2018). To further investigate whether ADARB1 expression is associated with the chemosensitivity of glioma cells, we used Spearman’s rank correlation method to examine the correlation between ADARB1 expression and TMZ sensitivity (IC50) in 5 glioma cell lines (U343, Hs683, U251, U118, and T98G). We observed a positive correlation between IC50 values and ADARB1 expression in these glioma cells (Spearman r = 0.960, *p* = 0.009) (Figure 2J). These results demonstrated that ADARB1 overexpression could promote the TMZ-resistance in GBM cells.

### ADARB1 was involved in AKT-Mediated TMZ Resistance of Glioma Cells

**FIGURE 3:**
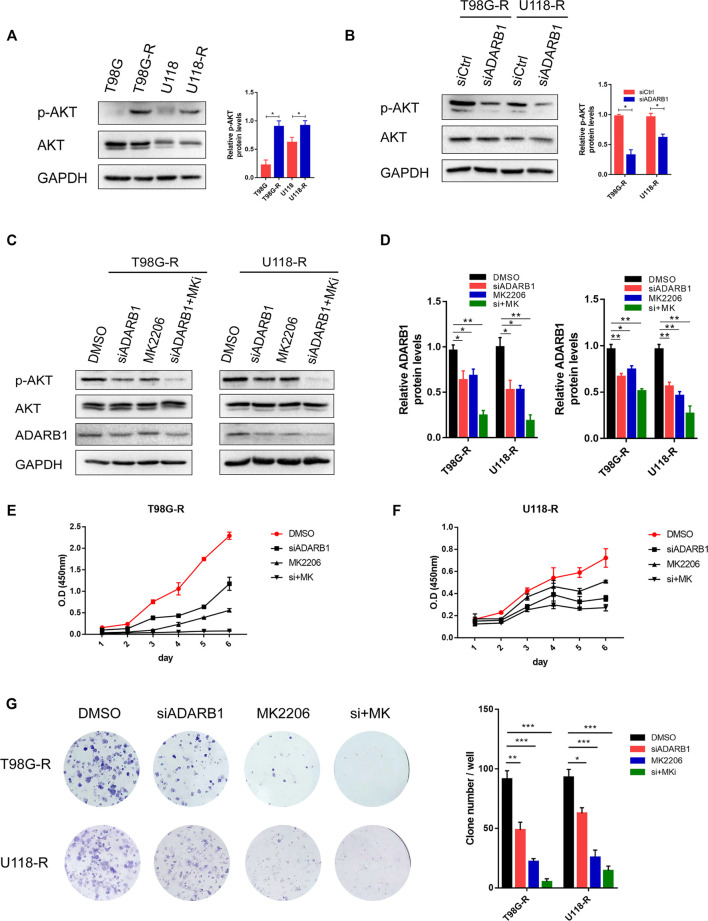
ADARB1 in AKT-mediated TMZ resistance of glioma cells. **(A)** Western blot for phospho-AKT (Ser-473) and AKT expression in T98G-R and U118-R cells. **(B)** Western blot for phospho-AKT (Ser-473) and AKT expression in T98G-R and U118-R cells treated with siCtrl or siADARB1. **(C,D)** Western blot for phospho-AKT (Ser-473), AKT, and ADARB1 in T98G-R and U118-R cells treated with DMSO, siADARB1, MK2206 and co-treated with siADARB1 and MK2206. **(E,F)** T98G-R and U118-R cells transfected with DMSO, siADARB1, MK2206 and co-treated with siADARB1 and MK2206 were subjected to MTS assays. **(G)** T98G-R and U118-R cells transfected with DMSO, siADARB1, MK2206 and co-treated with siADARB1 and MK2206 were subjected to colony formation assays.

Previous studies have shown that the AKT signaling pathway is important in the treatment of GBM (Shi et al., 2020; Zhong et al., 2020). Therefore, we explored whether ADARB1 mediates TMZ resistance through an AKT-dependent mechanism. First, we found that the expression level of p-AKT was significantly upregulated in TMZ-resistant glioma cell lines (T98G-R and U118-R cells) compared with their parental cell lines (T98G and U118 cells). This result indicated that AKT activation might display an important effect in the chemoresistance of GBM (Figure 3A). In contrast, knockdown of ADARB1 with siRNA significantly decreased the levels of p-AKT in T98G-R and U118-R cells (Figure 3B). To further identify the effect of the ADARB1-mediated AKT signaling axis in TMZ-resistant GBM cells, we treated TMZ-resistant glioma cells with the AKT inhibitor MK2206 (Mehnert et al., 2019). We observed that administration of MK2206 significantly inhibited the phosphorylation of AKT at Ser473 (Figures 3C,D). We also treated TMZ-resistant glioma cells with the combination of MK2206 and ADARB1 siRNA. The result showed that the inhibitory effect of the combined treatment on p-AKT expression was remarkably increased (Figures 3C,D). Moreover, cell proliferation assay and colony formation assay were also performed. We observed that combined treatment with MK2206 and siADARB1 on TMZ-resistant glioma cells resulted in a slower proliferation rate and lower clone number than the control or single treatment group (Figures 3E–G). The results above indicated that ADARB1 is involved in AKT-mediated TMZ resistance in GBM cells.

### Functional Enrichment Analysis of ADARB1-Associated Co-Expressed Genes

**FIGURE 4:**
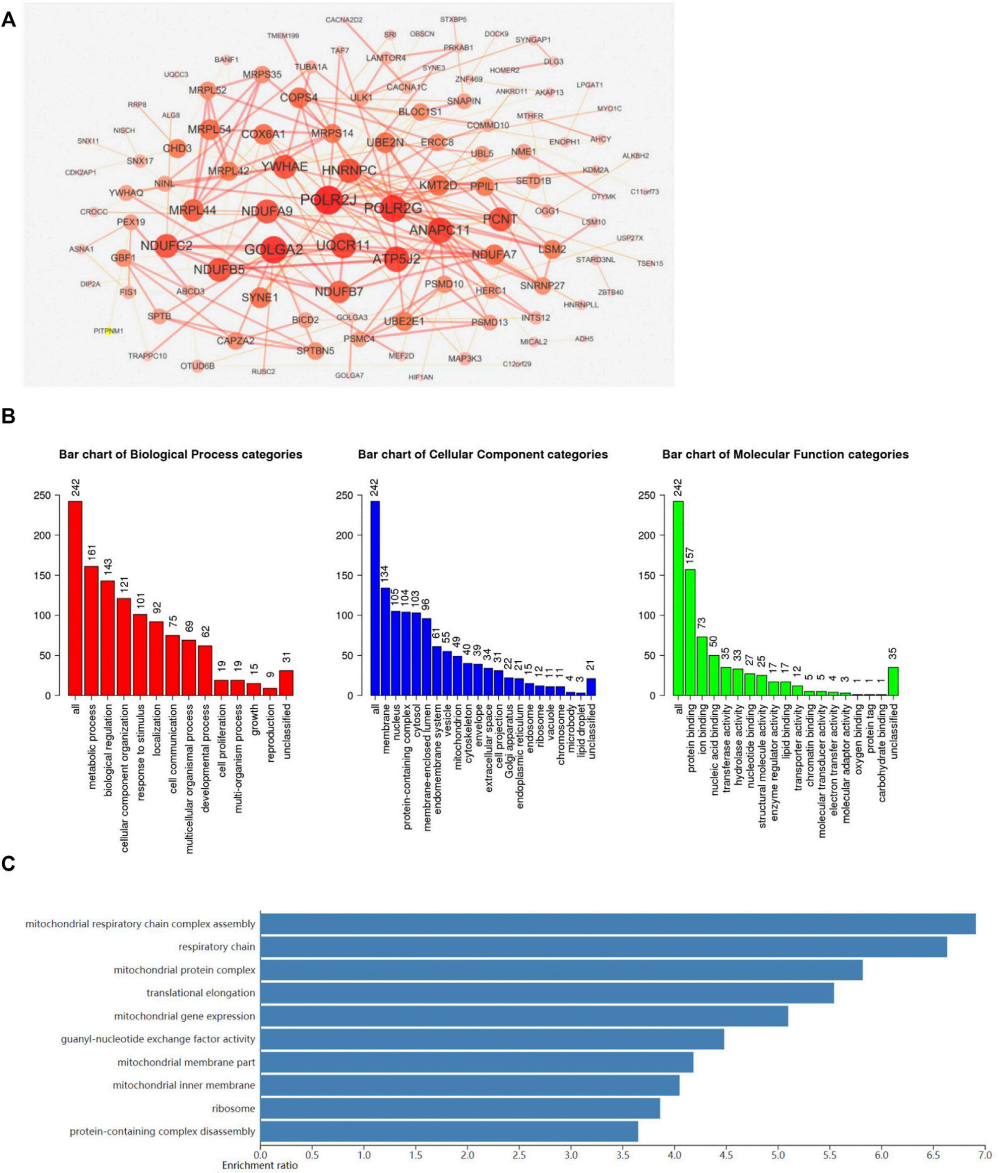
Functional enrichment analysis of ADARB1-associated co-expressed genes. **(A)** The PPI network of ADARB1 associated co-expressed genes was created by the STRING and Cytoscape software. **(B)** The GO analysis of ADARB1 associated co-expressed genes, including biological processes, cellular components and molecular function. **(C)** The KEGG pathway analysis of ADARB1 associated co-expressed genes.

To further investigate the potential role of ADARB1 in GBM pathogenesis, we performed a functional enrichment analysis of ADARB1 co-expressed genes using the cBioportal database (Supplementary Table S2). Studying the network of interactions between proteins can help uncover core regulatory genes and understand protein function (Athanasios et al., 2017). Thus, a PPI network of these co-expressed genes was studied using STRING and Cytoscape software (Figure 4A). In order to understand the underlying biological functions of these co-expressed genes, GO and KEGG analyses were conducted using the WebGestalt tool. The analysis of biological processes categories showed that these co-expressed genes were mainly related to metabolic processes and biological regulation. For the analysis of cellular components, co-expressed genes were mainly localized to cell membranes, nucleus, and protein-containing complex. Protein binding was significantly enriched for these co-expressed genes in molecular function categories (Figure 4B). In addition, the KEGG pathway demonstrated that these co-expressed genes were functionally enriched in several cancer-related signaling pathways, especially in the mitochondrial signaling pathway (Figure 4C).

### Regulation of Immune Molecules by ADARB1

**FIGURE 5:**
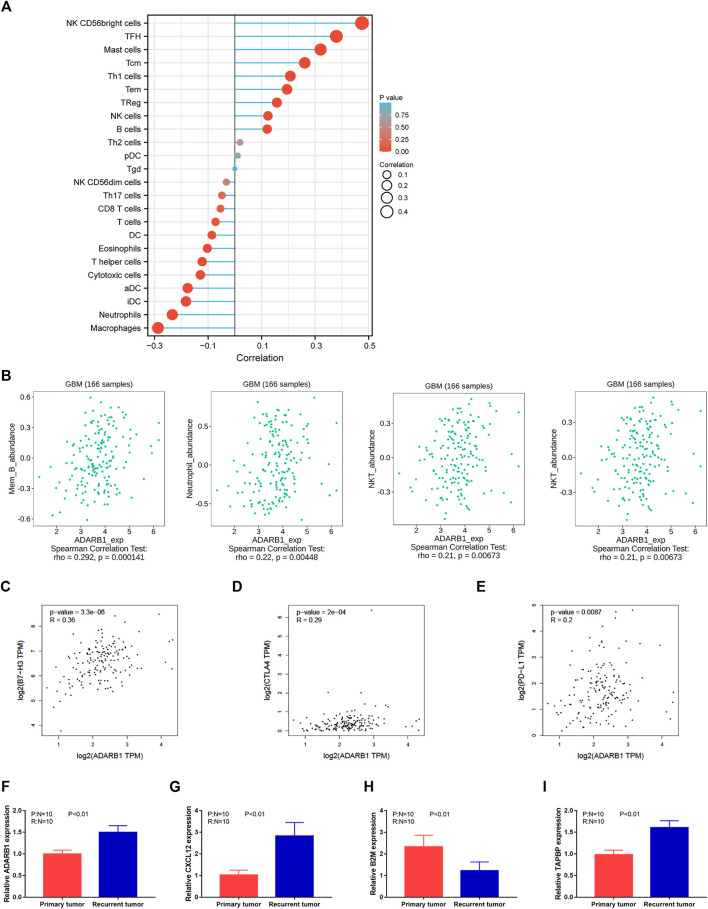
Regulation of immune molecules by ADARB1. **(A)** The bubble diagram analyzed by ssGSEA showing the correlation between ADARB1 and immune cells. **(B)** The correlation of ADARB1 expression with tumor-infiltrating lymphocytes (TILs) in GBM patients from TISDIB database. **(C–E)** The association between the expression level of ADARB1 and three immune checkpoints B7-H3, CTLA4 and PD-L1 from GEPIA database. **(F–I)** The expression of ADARB1 and immune molecules in primary and recurrent tumor of GBM patients using qPCR.

Previous studies reported that several immune pathways were associated with the pro-tumorigenic phenotypes of immune cells and protection of tumor cells from the immune attack, ultimately favoring the development and metastasis of tumors (de Almeida et al., 2020; Tsukioki et al., 2020). The distributions of immune cells related to ADARB1 expression are shown in the bubble diagram (Figure 5A) by ssGSEA analysis of Xiantao Tool (https://www.xiantao.love/products). Using the TISIDB database, we investigated the correlations between ADARB1 expression and immune-associated biomarkers, such as lymphocytes, immunomodulators, chemokine, and receptors. Figure 5B shows a positive correlation between ADARB1 expression and several tumor-infiltrating lymphocytes (TILs) in GBM patients. The lymphocytes exhibiting the most significant correlations are memory B cells (Spearman r = 0.292, *p* = 1.41e-04), NK cells (Spearman r = 0.326, *p* = 1.96e-05), Treg cells (Spearman r = 0.164, *p* = 0.0353), and NKT cells (Spearman r = 0.21, *p* = 6.73e-03).

Most immunomodulatory therapies have focused on increasing T-cell responses by targeting inhibitory pathways with immune checkpoint inhibitors or by targeting activating pathways. Although these approaches have led to prominent successes, only a minority of patients with cancer benefit from these treatments, highlighting the need to identify new cells and molecules that could be exploited in the next generation of immunotherapy (Demaria et al., 2019). Immunomodulators have been classified into three types, immunoinhibitors, immunostimulators, and major histocompatibility complex (MHC) molecules. Supplementary Figure S3A shows the correlation between ADARB1 expression and immunostimulators. The most significantly correlated immunostimulants include PVR (Spearman r = 0.292, *p* = 1.46e-04), CXCL12 (Spearman r = 0.393, *p* = 2.11e-07), IL6R (Spearman r = 0.217, *p* = 5.01e-03), and LTA (Spearman r = 0.181, *p* = 0.0199). Supplementary Figure S3B shows the correlations between ADARB1 expression and immunoinhibitors. The most significantly correlated immunoinhibitors include CSF1R (Spearman r = 0.153, *p* = 0.0493), LAG3 (Spearman r = 0.244, *p* = 1.61e-03), CD160 (Spearman r = 0.274, *p* = 3.71e-04) and CTLA4 (Spearman r = 0.179, *p* = 0.0212). Supplementary Figure S3C shows the correlations between ADARB1 expression and MHC molecules. And the MHC molecules showing the most significant correlations are B2M (Spearman r = -0.245, *p* = 1.54e-03), TAP2 (Spearman r = 0.2, *p* = 0.01), TAPBP (Spearman r = 0.303, *p* = 7.65e-05), and HLA-DMB (Spearman r = -0.154, *p* = 0.0474).

As molecular messengers, cytokines allow immune system cells to communicate with each other to produce coordination of target antigens, it have regulatory and effector functions in many diseases, thus cytokines and their receptors can be used in immunotherapy (Conlon et al., 2019). Supplementary Figure S4A shows the correlation between ADARB1 expression and chemokines. The chemokines showing the most significant correlations are CCL28 (Spearman r = 0.23, *p* = 2.96e-03), CXCL12 (Spearman r = 0.393, *p* = 2.11e-07), CXCL1 (Spearman r = 0.171, *p* = 0.0276) and CX3CL1 (Spearman r = 0.221, *p* = 4.38e-03). Supplementary Figure S4B shows the correlation between ADARB1 expression and chemokine receptors. The receptors displaying the most significant correlations are CCR5 (Spearman r = 0.178, *p* = 0,219), CXCR1 (Spearman r = 0.172, *p* = 0.027), CCR10 (Spearman r = -0.169, *p* = 0.0299), and CCR6 (Spearman r = 0.214, *p* = 5.7e-03).

Immune checkpoint inhibitors (ICIs) have developed in the field of tumor therapy. and ICIs are now first-line therapies for various solid and liquid tumors (Bagchi et al., 2021). Furthermore, the data collected from the GEPIA database show that three immune checkpoints, B7-H3, CTLA4, and PD-L1, are positively correlated with ADARB1 expression (Figures 5C–E). Based on these results, we speculated that ADARB1 might have a stronger effect on immune fingerprinting in GBM patients.

We also verified the expression of several immune molecules in TMZ response and resistant human GBM tissues. As shown in Figures 5F–I, we found that the expression level of ADARB1, CXCL12 and TABPB were higher in recurrent tumor tissues, whereas the expression level of B2M was lower in recurrent tumor tissues.

## Discussion

TMZ resistance in GBM is still a major clinical issue in its treatment (Ni et al., 2020). Our study aimed to explore the underlying mechanisms of ADARB1 functions in TMZ resistance of GBM. Intriguingly, we found that ADARB1 was significantly upregulated in GBM tissues and cell lines, and involved in AKT-mediated TMZ resistance. Based on the bioinformatic analysis, ADARB1 was also found to be associated with immune-related molecules. Aberrant expression of ADARB1 might play an important role in immune escape and can be an immunotherapeutic target for GBM.

A-to-I RNA editing is the most abundant RNA editing mediated by ADAR enzymes in mammalian tissues (Patil et al., 2020). It has been shown that in many human cancers, including GBM, dysregulated levels of RNA editing were frequently occurred (Seeburg, 2002). In GBM, ADARB1 has been extensively reported as a tumor suppressor gene. Surprisingly, we found that ADARB1 expression was significantly upregulated in TMZ-resistant glioma cells. These conflicting results might be due to chemotherapy-induced alterations in gene expression (35).

AKT is a serine/threonine kinase activated by a dual regulatory mechanism that requires translocation to the plasma membrane and phosphorylation (Wu et al., 2020b; Ke et al., 2020). High levels of p-AKT expression have been reported to associate with poor prognosis in GBM patients (Duggan et al., 2021). Studies have reported that activation of AKT has an important impact on ADARB1-dependent RNA editing, which is involved in cell maintenance and development (Piazzi et al., 2020). AKT has been found to directly promote ADARB1 phosphorylation, thereby regulating multiple signaling pathways associated with cell survival (Bavelloni et al., 2019). In addition, Chen and colleague have found that ADARB1 overexpression could induce insulin-like growth factor binding protein 7-dependent inhibition of AKT signaling (Chen et al., 2017). In our study, we found the expression level of p-AKT significantly decreased in TMZ-resistant glioma cells after ADARB1 knockdown. Besides, a combined treatment with AKT inhibitor and ADARB1 knockdown obviously resulted in much slower proliferation rates of TMZ-resistant glioma cells, indicating that ADARB1 might be involved in AKT-mediated TMZ resistance of glioma cells.

Due to the limited efficacy of existing therapies, immunotherapy is being widely investigated in patients with GBM. In recent studies, increasing evidence has indicated that tumor microenvironments and immune infiltration are important in tumor development and chemoresistance (Ju et al., 2020a; Ju et al., 2020b). Immunotherapy has emerged as a powerful method for tumor treatment, which attacks and kills the tumor cells by stimulating the body’s immune system to recognize tumor cells and activate specific immune cells (Demircan et al., 2020). Meanwhile, immunotherapy combined with conventional treatments, such as surgery, chemotherapy, and radiation, represents a promising approach (Jia et al., 2020). In this study, the correlation between ADARB1 and the immune system was assessed with the TISIDB database. The results revealed that ADARB1 had the highest correlation with multiple TILs, including memory B, NK, Treg, and NKT cells. Besides, ADARB1 had the most significant correlations with immunostimulators, immunoinhibitors, and MHC molecules, such as CXCL12, TABPB and B2M. Moreover, in GBM patients, the chemotherapy response status was based on progression-free survival after TMZ treatment, named as TMZ resistance (recurrence within 4 months after surgical resection) and TMZ response (no recurrence within 4 months after surgical resection) (Yan et al., 2019). Accordingly, we verified the expression of several immune molecules in TMZ response and resistant GBM tissues. We found that the expression level of ADARB1, CXCL12 and TABPB were higher in recurrent tumor tissues, whereas the expression level of B2M was lower in recurrent tumor tissues. Additionally, three immune checkpoints, B7-H3, CTLA4, and PD-L1, were positively correlated with ADARB1 expression in GBM patients. These findings suggest that ADARB1 may play a very important role in the immune regulation in GBM patients and can be a potential immunotherapeutic target. However, the underlying mechanisms remain to be fully explained.

Emerging studies have demonstrated the important roles of mitochondrial dysfunction in TMZ resistance of GBM patients. TMZ resistance in GBM has been proved to be associated with increased mitochondrial coupling and decreased concentration of reactive oxygen species (Oliva et al., 2011). Regulation of mitochondrial apoptosis signaling could sensitize U87 GBM cells to temozolomide treatment (He et al., 2018). In our study, the KEGG analysis of ADARB1-associated co-expressed genes indicated that ADARB1 was potentially involved in several mitochondrial processes, including mitochondrial respiratory chain complex assembly, mitochondrial protein complex, etc. Therefore, these findings suggested ADARB1 might promote the TMZ resistance through modulating the mitochondrial functions in GBM.

In conclusion, this study reports the association of the RNA editing gene ADARB1 with the TMZ chemoresistance of GBM for the first time. Aberrant expression of ADARB1 is a promising biomarker for the prognosis of GBM patients. Furthermore, ADARB1 is found to be involved in AKT-mediated TMZ resistance in glioma cells. Therefore, ADARB1 may play an important role in the therapeutic response and immune regulation of GBM.

## Data Availability

The original contributions presented in the study are included in the article/Supplementary Material, further inquiries can be directed to the corresponding authors.

## Supplementary Material

Supplementary Figure S1 The profiles of ADAR1 in GBM patients. (A-B) The mRNA expression of ADAR1 in GBM and noncancerous tissues were analyzed from two datasets, GSE4536 and GSE4290. Data performed with mean ± SD subjected to Student’s t-test. (C) The relationship between ADARB1 expression and OS analyzed from GSE13041 dataset.

Supplementary Figure S2 The effect of ADARB1 knockdown on the parental cells. (A-B) T98G and U118 cells transfected with siADARB1 were subjected to MTS assays and colony formation assays.

Supplementary Figure S3 Correlation of ADARB1 expression with immunostimulators, immunoinhibitors, and MHC molecules in GBM. (A) The correlation between ADARB1 expression and immunostimulators. And the top four immunostimulators showing the most significant correlation with ADARB1 expression. (B) The correlation between ADARB1 expression and immunoinhibitors. And the top four immunoinhibitors showing the most significant correlation with ADARB1. (C) The correlation between ADARB1 expression and MHC molecules. And the top four MHC molecules showing the most significant correlation with ADARB1.

Supplementary Figure S4 Correlation of ADARB1 expression with chemokines and receptors in GBM. (A) The correlation between ADARB1 expression and chemokines. And the top four chemokines showing the most significant correlation with ADARB1 expression. (B) The correlation between ADARB1 expression and receptors. And the top four receptors showing the most significant correlation with ADARB1.
