# Supplementary figures and images for: Aberrant Expression of ADARB1 Facilitates Temozolomide Chemoresistance and Immune Infiltration in Glioblastoma
Source: Front Pharmacol. 2022 Feb 1;13:768743. doi: 10.3389/fphar.2022.768743 (PMC8844449; doi:10.3389/fphar.2022.768743)

Figure S2

A

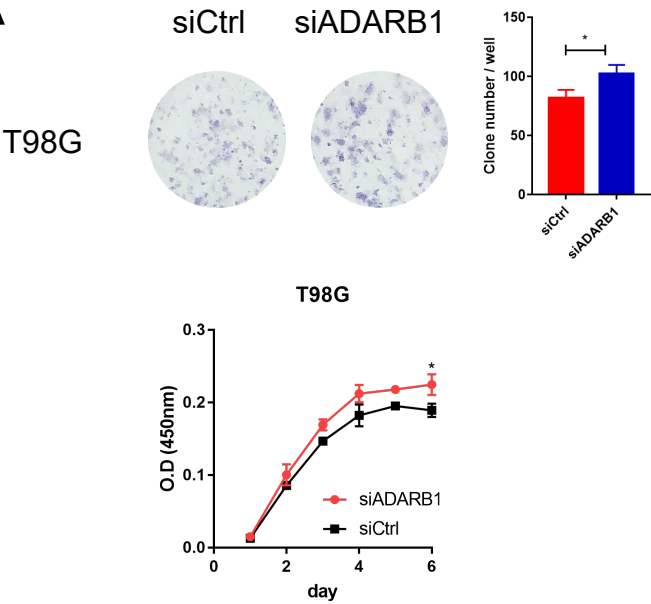

B

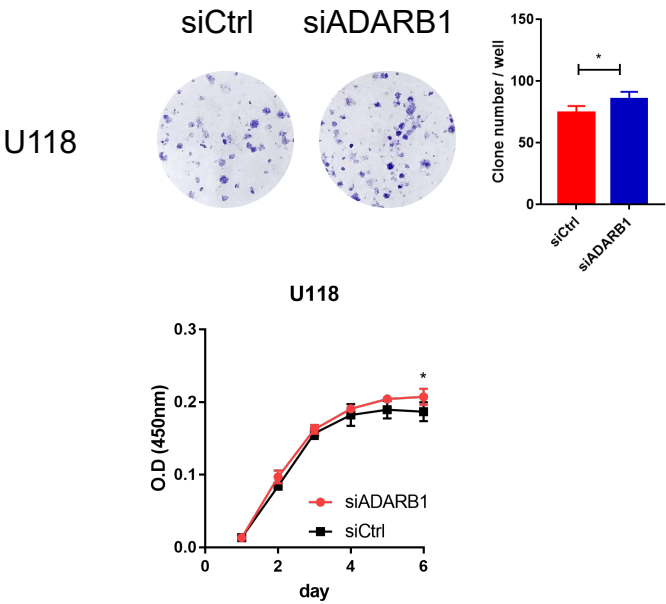

Supplement: Supplementary file 1 [file DataSheet2.PDF]

## Figure S4

A

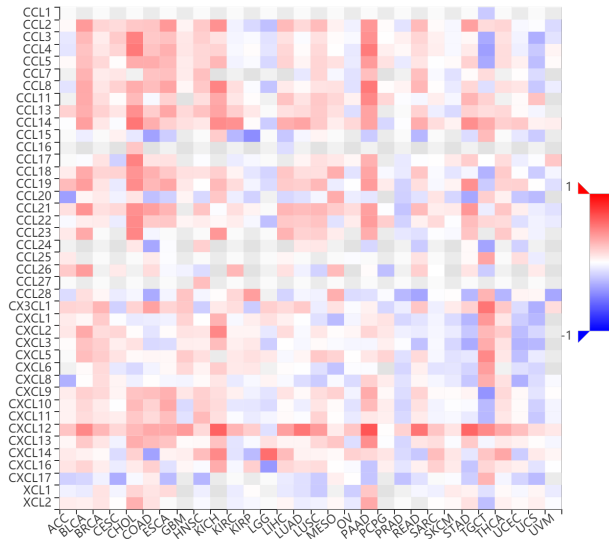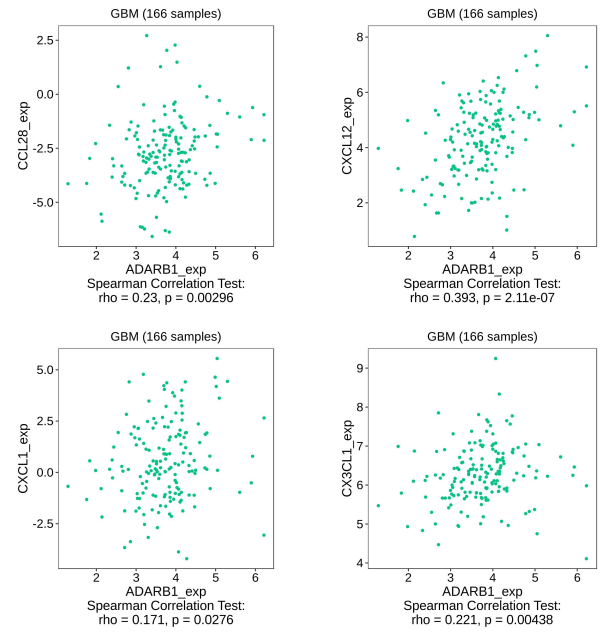

# B

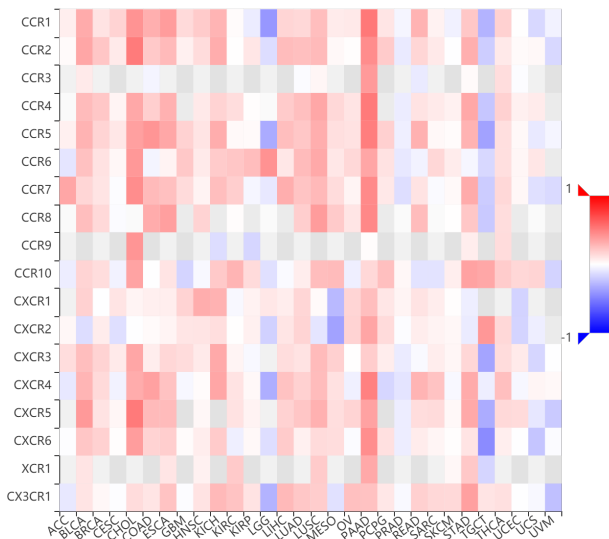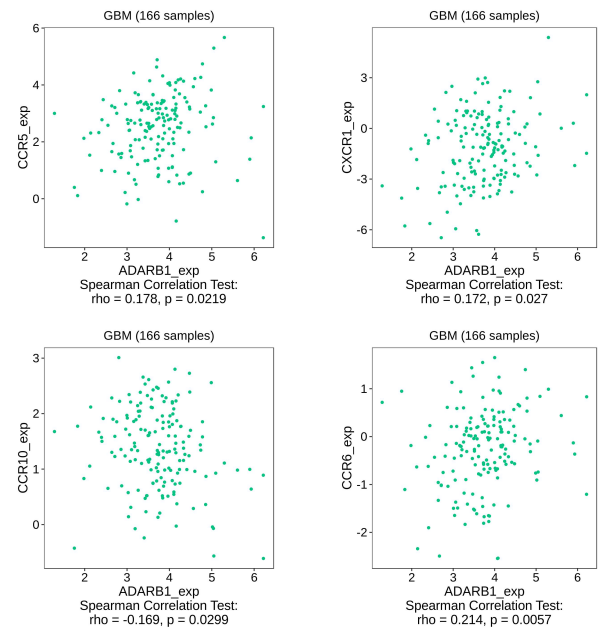

Supplement: Supplementary file 3 [file DataSheet4.PDF]

A

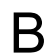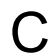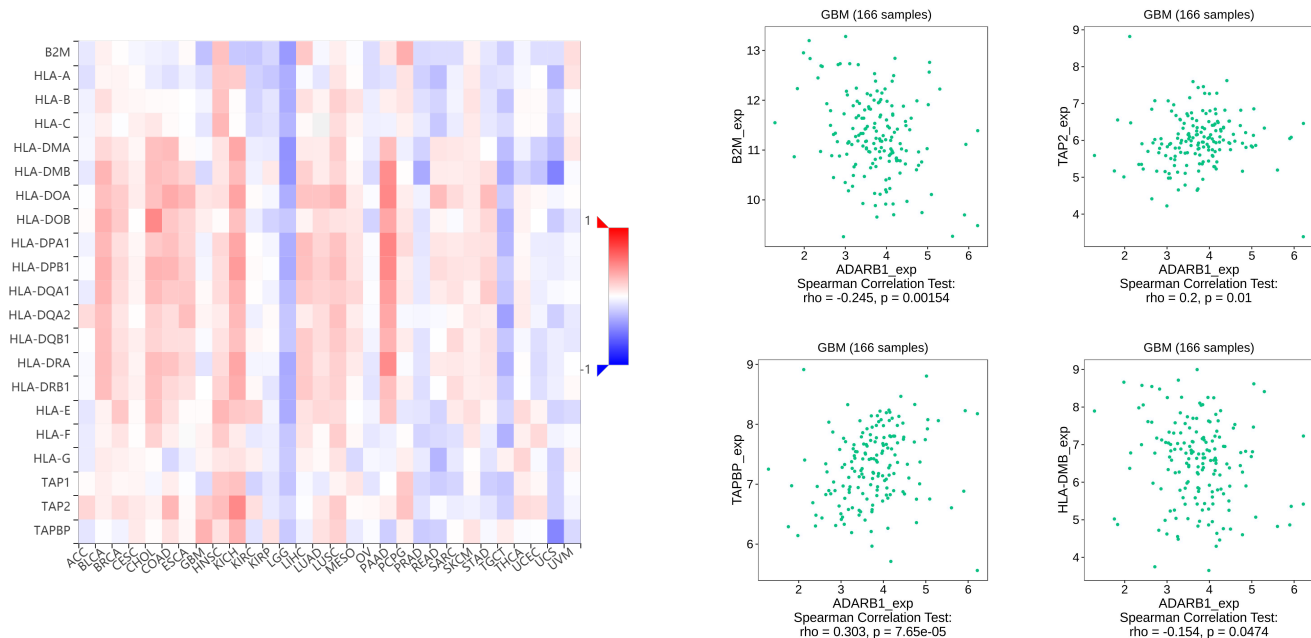

Supplement: Supplementary file 5 [file DataSheet3.PDF]

Figure S1

A GSE4536

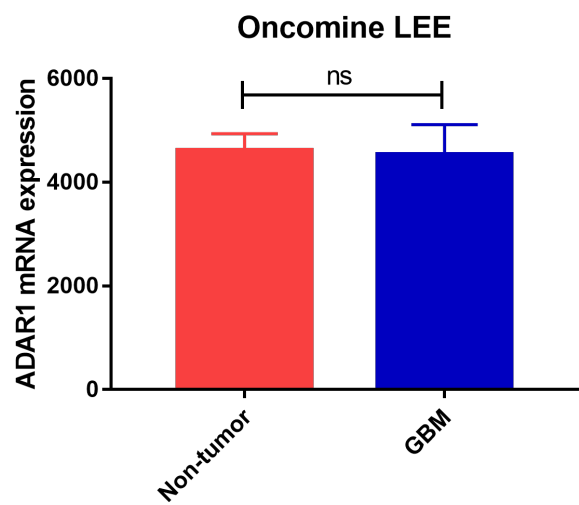

B GSE4290

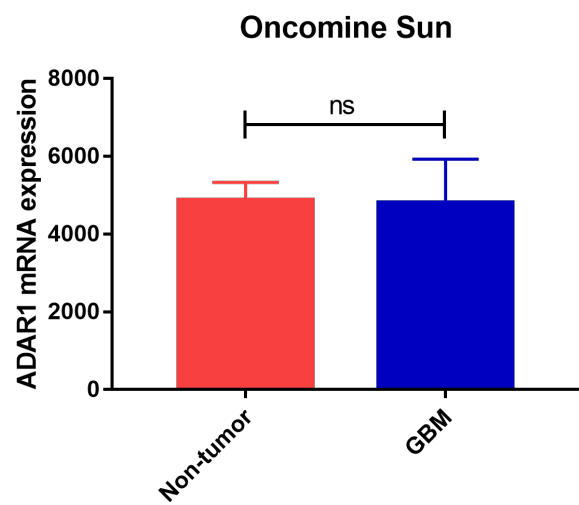

C GSE13041

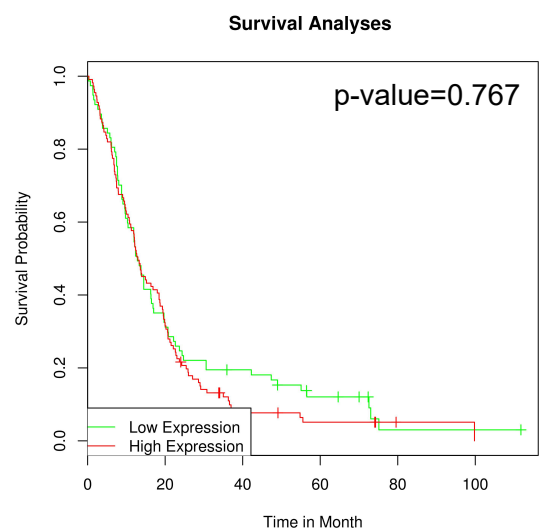

Supplement: Supplementary file 6 [file DataSheet1.PDF]
