# Supplementary material for: Aberrant Expression of ADARB1 Facilitates Temozolomide Chemoresistance and Immune Infiltration in Glioblastoma
Source: Front Pharmacol. 2022 Feb 1;13:768743. doi: 10.3389/fphar.2022.768743 (PMC8844449; doi:10.3389/fphar.2022.768743)
Supplement: Supplementary file 4 [file Table1.docx]

**Table S1.The main bioinformatics databases used to analyze the role of ADARB1 in GBM.**

| **Database** | **URL** | **Refs.** |
| --- | --- | --- |
| Oncomine | https://www.oncomine.com/resurce/login.html | （18） |
| cBioPortal | http://www.cbioportal.org/ | （25） |
| Drugsurv | http://www.bioprofiling.de/drugsurv | （33） |
| PrognoScan | http://gibk21.bse.kyutech.ac.jp/PrognoScan/index.html | （34） |
| STRING | http://string-db.org/ | （26） |
| Cytoscape | http://cytoscape.org/ | （28） |
| TISIDB | http://cis.hku.hk/TISIDB | （30） |
