# Supplementary material for: Aberrant Expression of ADARB1 Facilitates Temozolomide Chemoresistance and Immune Infiltration in Glioblastoma
Source: Front Pharmacol. 2022 Feb 1;13:768743. doi: 10.3389/fphar.2022.768743 (PMC8844449; doi:10.3389/fphar.2022.768743)
Supplement: Supplementary file 7 [file DataSheet5.PDF]

CERTIFICATE OF ENGLISH EDITING

This document certifies that the manuscript entitled

"Aberrant expression of ADARB1 facilitates temozolomide chemoresistance and immune infiltration in glioblastoma"

was edited for English language, including grammar, punctuation and spelling by one or more native English-speaking editors of KetengEdit. Neither the research content nor the authors' intentions were altered in any way during the editing process.

Best Regards

Keteng Editing Team

Date Issued

October 20, 2021

Disclaimer: The changes in the document may be accepted or rejected by the authors in their sole discretion after our editing. However, KetengEdit is not responsible for revisions made to the document after our edit on October 18, 2021.

To verify the final edited version, or if you have any questions or concerns regarding the edited document, please contact KetengEdit at fudan1392@163.com. For more details regarding our company and current services, please visit: http://www.ketengedit.com

---
